# Profiling of volatile flavor compounds in *nkui* (a Cameroonian food) by solid phase extraction and 2D gas chromatography time of flight mass spectrometry (SPME‐GC×GC‐TOF‐MS)

**DOI:** 10.1002/fsn3.736

**Published:** 2018-10-06

**Authors:** Oluwafemi A. Adebo, Patrick B. Njobeh, Steve C. Z. Desobgo, Mark Pieterse, Eugenie Kayitesi, Derek T. Ndinteh

**Affiliations:** ^1^ Faculty of Science Department of Biotechnology and Food Technology University of Johannesburg Johannesburg South Africa; ^2^ Faculty of Science Department of Applied Chemistry University of Johannesburg Johannesburg South Africa; ^3^ LECO Africa Pty Ltd Johannesburg South Africa

**Keywords:** flavor, GC×GC‐TOF‐MS, *nkui*, SPME

## Abstract

The objective of this study was to investigate the volatile flavor compounds of *nkui*, a Cameroonian food, using solid phase microextraction (SPME) and a two‐dimensional gas chromatography time of flight mass spectrometry GC×GC‐TOF‐MS system. Using SPME, volatile compounds were extracted from *nkui* and analyzed by GC×GC‐TOF‐MS. The data retrieved revealed the presence of flavor volatiles including acids (20%), alcohols (4%), aldehydes (10%), aromatic compounds (4%), esters (7%), furans (4%), ketones (11%), terpenes and terpernoids (27%). Although the terpene compounds were the most predominant, an ester (linalyl acetate) had the highest percentage of 19%, conferring a sweet, green and citrus flavor. Results obtained from this study suggest that the characteristic flavor of *nkui* was due to the combination of different volatile flavor compounds, which contributed to its aroma. Considering the medicinal importance of these compounds, their presence positions *nkui* as a vital food source with health benefits and medicinal properties.

## 1 INTRODUCTION

Plant parts are frequently used as spices, food additives, and meals. *Nkui* is a traditional heavily spiced Cameroonian soup, consumed as food and utilized in traditional medicine for nursing mothers. It is made from the combination of different stems and spices including *Afrostyrax lepidophyllus* Mildbr., *Capsicum frutescens* Linn., *Fagara leprieurii* Guill. et Perr., *Fagara tessmannii* Engl., *Mondia whitei* Hook. F. Skell., *Pentadiplandra brazzeana* Baill., *Solanum gilo* Raddi., *Tetrapleura tetraptera* Thaub., and *Xylopia parviflora* A. Rich. Benthane (Tchoupang et al., 2016). *Nkui* has a rich, distinct, and pleasant flavor, perceived during consumption and attributed to its abundant volatile compounds.

Flavor is a major factor that determines consumer selection, perception, acceptance of a particular food product and thus plays a significant role in the food. The flavor of any food is thus largely dependent on the number, quantity, and characteristics of the different volatile compounds it contains (Jelen, Majcher, & Dziadas, 2012). Such is extended to food substances and subsequent preparations from them. Despite the rich flavor and other volatile components present in *nkui*, previous studies that investigated *nkui* for these compounds have not been presented in the literature.

A comprehensive understanding and knowledge of volatile compounds in foods is quite complex and requires an efficient extraction and subsequent analytical technique. While several extraction techniques including solid phase extraction (SPE), stir bar sorptive extraction (SBSE), pressured hot water (PHW), and liquid–liquid extraction (LLE) are known (Gbashi, Adebo, Piater, Madala, & Njobeh, 2017; Goncalves et al., 2016), challenges around cost, environmental friendliness, ease of extraction, and cross‐contamination are their major challenges. The emergence of solid phase microextraction (SPME) has provided an efficient, robust, selective, cost effective, and environmentally friendly extraction technique and their combination with an effective detection technique offer enormous potentials (George et al., 2018; Kusano, Kobayashi, Iizuka, Fukushima, & Saito, 2016). The effectiveness of SPME is also demonstrated in its ability to integrate extraction, concentration, and analyte injection into a single process, ensuring sample throughput (Goncalves et al., 2016).

GC×GC‐TOF‐MS is sensitive chromatographic technique for both separation and detection of compounds. Its capabilities with includes increased mass spectral identification and deconvolution algorithms, identification abilities, enabled detection of thousands of peaks and the provision of additional data makes it a suitable analytical platform. Its compatibility with SPME for profiling of volatile flavor compounds has been reported (Ding, Wu, Huang, & Zhou, 2016; Goncalves et al., 2016). The aim of this study was thus to investigate the flavor components in *nkui* using SPME and subsequent analysis on GC×GC‐TOF‐MS.

## 2 MATERIALS AND METHODS

### 2.1 *Nkui* sampling and composition

Different plant materials that make up *nkui* were collected, identified by the National Herbarium in Cameroon, and deposited as specimens. The specimens were issued voucher numbers and the different plant parts and quantities used to make up the *nkui* are presented in Table 1.

**Table 1:** Composition and proportion of materials making up *nkui*

No.	Scientific name	Local name	Specimen no.	Part used	Quantity (g)
1	*Afrostyrax lepidophyllus* Mildbr. (Huaceae)	*Mbadum*	31115 HNC	Fruit	5 (5.55)^a^
2	*Capsicum frutescens* Linn. (Solanaceae)	*Soga*	34726 HNC	Fruit	1 (1.11)
3	*Fagara leprieurii* Guill. et Perr. (Rutaceae) syn. *Zanthoxylum leprieurii* Guill. et Perr.	*Manyanje*	42992 HNC	Fruit	3 (3.33)
4	*Fagara tessmannii* Engl. (Rutaceae)	*Nga'ncu*	38960 HNC	Fruit	7 (7.78)
5	*Mondia whitei* Hook. F. Skell. (Pleriplocaceae)	*Dmte*	34180 HNC	Root	35 (38.89)
6	*Pentadiplandra brazzeana* Baill. (Pentadiplandraceae)	*Ndu'fe*	41536 HNC	Root	11 (12.22)
7	*Solanum gilo* Raddi. (Solanaceae)	*Seban*	14602	Fruit	6 (6.66)
8	*Tetrapleura tetraptera* Thaub. (Leguminosae)	*Dumnkag*	31310 HNC	Fruit	2 (2.22)
9	*T. tetraptera* Thaub. (Leguminosae)	*Kubdum*	31310 HNC	Stem bark	19 (21.11)
10	*Xylopia parviflora* A. Rich. Benthane (Annonaceae)	*Mbatu'u*	42349 HNC	Fruit	1 (1.11)

a Values in parentheses represent percentages.

### 2.2 SPME sampling

The extraction of *nkui* flavor components was done using a 50/30 μm SPME fiber coated with divinylbenzene/carboxen/polydimethylsiloxane (DVB/CAR/PDMS) (Supelco, Inc., Bellefonte, PA). Briefly, 20 g of the sample was placed in a head‐space vial heated at 40°C for 20 min. Sampling was then done by exposing the fiber to the headspace of the sample for 20 min, after which the SPME device was transferred to the GC×GC‐TOF‐MS equipment for analysis. The process was repeated four times, though before each analysis the fibers were thermally cleaned and conditioned by heating them at 270°C in a stream of helium. A SPME fiber (Supleco, South Africa) was used to sample the volatile compound and immediately injected into a GC‐MS system for analysis.

### 2.3 GC×GC‐TOF‐MS analysis

Solid phase microextraction analyte samples were analyzed using a LECO Pegasus 4D Time of Flight mass spectrometer (LECO Corporation, St Joseph, MI, USA) equipped with a modified Agilent 7890A Gas Chromatograph (Agilent Technologies, Inc., Wilmington, DE, USA), a LECO GC×GC modulator and secondary oven (LECO Corporation, St Joseph, MI, USA) and a split/splitless inlet. The columns set used were: Rxi‐5 SilMS (29.5 m × 0.25 mm × 0.25 μm) as a primary column and Rxi 17 Sil MS (0.95 m × 0.25 mm × 0.25 μm) as the secondary column (Restek, Bellefonte, PA, USA). Helium was used as a carrier gas at a constant flow rate of 1 ml/min and an inlet temperature of 250°C. An initial oven temperature of 40°C was set and held for 0.5 min and then slowly ramped at 10°C/min to 250°C and then held for 0.5 min at 250°C. The modulator and secondary oven were run at an offset temperature of 5°C above the primary oven. The mass spectrometer was set up under the following conditions: no solvent delay because it was a SPME analysis; transfer line temperature at 250°C; Electron ionization at −70 eV; source temperature at 250°C; stored mass range: 45–600 μ; acquisition rate: 10 spectra/s for GC×GC‐TOF‐MS; detector offset voltage was set at 300 V.

Retention time alignment, matched filtration, peak detection, and peak matching were done on ChromaTOF software (LECO, USA). Subsequent identification was done by comparison with mass spectral databases (NIST, Adams, and EO libraries). A semi quantification of each compound was calculated on the basis of peak areas and relative concentration presented in %.

### 2.4 Statistical analysis

Average of the replicate data obtained from the GC×GC‐TOF‐MS analysis was computed and the result presented as mean ± standard deviation.

## 3 RESULTS AND DISCUSSION

**Figure 1:**
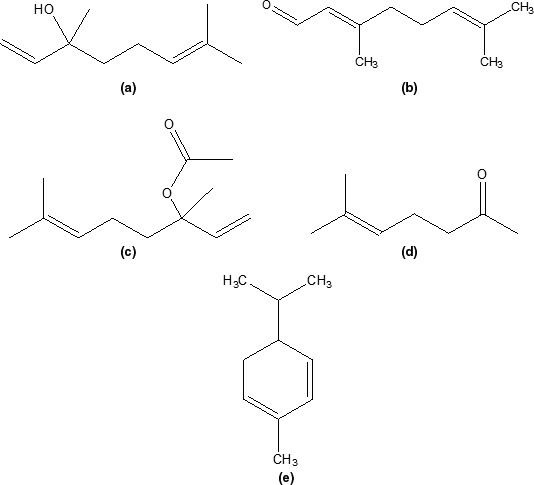
Major compounds in *nkui*: (a) Linalool, (b) 2,6‐octadienal,3,7‐dimethyl, (c) Linalyl acetate, (d) 6‐methyl‐5‐hepten‐2‐one, (e) à‐phellandrene

Although a large number of volatile compounds are in foods, only a fraction of them eventually enhance flavor and aroma, as the perception of these sensory qualities is mainly driven by a combination of active volatile compounds sensed in the retronasal and/or orthonasal cavity (Bertuzzi, McSweeney, Rea, & Kilcawley, 2018). As these compounds have different characteristics, their concentrations can also affect the aroma of the finished food product. To identify the significant compounds that contribute to the flavor of *nkui*, this study investigated its flavor profile using SPME‐GC×GC‐TOF‐MS. Although a total of 250 compounds were obtained from the GC profiles, only 92 flavor related compounds were identified and subsequently divided into groups. This consisted of acids (8), alcohols (14), aldehydes (5), aromatic compounds (9), esters (9), furans (4), ketones (12), sulfur compounds (2), terpenes/terpenoids (22), and miscellaneous compounds (2) (Table 2). The structures of the major flavor compounds identified in the *nkui* are provided in Figure 1.

**Table 2:** Volatile flavor compounds identified in *nkui* by SPME‐GC×GC‐TOF‐MS, their group classification and flavor descriptors

Name	RT (s)	Quantity (%)	Flavor descriptors^a^
Acids			
Butanoic acid	692.6	0.002 ± 0.00	Butter, acidic, fruity, rose
Acetic acid	716.6	1.712 ± 0.79	Vinegar, sour, pungent
Propanoic acid	806.7	0.002 ± 0.00	Acidic, diary, fruity
3,3‐dimethylacrylic acid	857.5	0.002 ± 0.02	Green, phenolic, diary
Butanoic acid, 3‐methyl‐	929.9	0.002 ± 0.00	Cheese, dairy, creamy
Hexanoic acid	1,077.3	0.009 ± 0.00	Fatty, cheesy, fruity
Hexanoic acid, 2‐ethyl‐	1,160.8	0.003 ± 0.00	Oily rancid, sweat‐like
Nonanoic acid	1,317.1	0.004 ± 0.00	Waxy, fatty cheesy
Alcohols			
1‐octen‐3‐ol	728.8	0.035 ± 0.01	Sweet, Mushroom, earthy, fungal
4‐thujanol, stereoisomer	739.5	0.224 ± 0.31	Cooly, minty
Bicyclo[3.1.1]hept‐3‐en‐2‐ol, 4,6,6‐trimethyl‐, [1s‐(1à,2á,5à)]‐	792.7	0.069 ± 0.05	Pine, ozone
Linalool	827.9	17.849 ± 7.18	Citrus, orange, floral
1,2‐propanediol	856.4	0.028 ± 0.02	Odorless/fatty aroma
1,5,7‐octatrien‐3‐ol, 3,7‐dimethyl‐	882.1	0.021 ± 0.02	Moldy
Terpinen‐4‐ol	896.0	0.159 ± 0.09	Woody, earthy, musty
Pinocarveol	918.9	0.012 ± 0.01	Camphoreous, pine, woody
*Trans*‐piperitol	942.2	0.067 ± 0.05	Herbal
*Trans*‐carveol	948.2	0.028 ± 0.03	Caraway, solvent, spearmint
Farnesol	972.9	0.013 ± 0.04	Floral, juicy, green
2,6‐octadien‐1‐ol, 3,7‐dimethyl‐, (E)‐	1,084.0	4.438 ± 5.98	Floral
Nerolidol	1,233.3	0.022 ± 0.01	Green, floral, fruity
Thymol	1,341.4	0.002 ± 0.00	Medicinal spicy
Aldehydes			
Nonanal	664.6	0.053 ± 0.03	Green, fat, citrus
Bicyclo[3.1.1]hept‐2‐ene‐2‐carboxaldehyde, 6,6‐dimethyl‐	891.1	0.031 ± 0.01	Spicy, herbaceous
2,6‐octadienal, 3,7‐dimethyl‐	941.1	0.139 ± 0.01	Lemon
Cinnamaldehyde	1,064.0	0.008 ± 0.00	Cinnamon, spicy
Lilac aldehyde D	1,151.9	0.069 ± 0.03	Floral, lilac
Aromatic compounds			
Benzene, 1‐methyl‐4‐(1‐methylethyl)‐	516.6	8.212 ± 9.10	Spicy, balsamic, musty
Benzene, 1‐methyl‐2‐(1‐methylethyl)‐	519.2	1.764 ± 0.08	Green, rubber
Benzene, 1‐methyl‐4‐(1‐methylethenyl)‐	708.7	0.077 ± 0.02	Spicy, balsamic, musty
Benzaldehyde	790.3	0.031 ± 0.01	Nutty, bitter, woody
Linalyl anthranilate	839.9	13.372 ± 0.11	Fresh, linalool, orange, blossom
Benzaldehyde, 4‐(1‐methylethyl)‐	1,023.4	0.025 ± 0.00	Nutty, bitter
Benzenemethanol, à,à,4‐trimethyl‐	1,081.3	0.046 ± 0.04	Sweet, fruity, cherry
Quinoxaline, 5‐methyl‐	1,169.0	0.014 ± 0.01	Nutty, peanut, roasted
Benzenemethanol, 4‐(1‐methylethyl)‐	1,270.1	0.007 ± 0.01	Cumin, spicy, floral
Esters			
Ethyl acetate	129.3	0.783 ± 0.03	Fruity, brandy‐like
Isononyl acetate	674.8	0.004 ± 0.00	Herbal, woody
(Z)‐3‐hexenyl acetate	701.7	0.015 ± 0.08	Green, fruity, apple pear
1,6‐octadien‐3‐ol, 3,7‐dimethyl‐, acetate	837.4	10.345 ± 8.59	Bergamot, lavender
Linalyl acetate	840.3	19.088 ± 0.12	Sweet, green, citrus
Terpinyl propionate	887.1	1.501 ± 1.12	Floral, lavender
Geranyl formate	968.3	0.079 ± 0.00	Green floral
Neryl acetate	987.1	0.849 ± 0.04	Green, citrus like
2,6‐octadien‐1‐ol, 3,7‐dimethyl‐, acetate	1,013.1	2.344 ± 1.54	Floral, rosy, sweet
Furan compounds			
Furanoid	715.5	0.320 ± 0.02	Earthy, floral, sweet, woody
2‐furanmethanol, 5‐ethenyltetrahydro‐à,à,5‐trimethyl‐, *cis*‐	744.6	0.725 ± 1.33	Earthy, floral, sweet
Rosefuran epoxide	875.7	0.010 ± 0.01	Green, earthy, citrus
Furan, 2‐ethyl‐5‐methyl‐/2‐ethyl‐5‐methylfuran	1,260.1	0.006 ± 0.01	Gassy, burnt
Ketones			
6‐methyl‐5‐hepten‐2‐one	597.9	0.632 ± 0.37	Green, vegetable, musty, mushroom
2‐nonanone	659.1	0.464 ± 0.04	Fruity, herbaceous
Bicyclo[2.2.1]heptan‐2‐one, 1,7,7‐trimethyl‐, (1s)‐	781.4	0.032 ± 0.04	Camphoreous
4‐isopropylcyclohex‐2‐en‐1‐one	800.2	0.017 ± 0.01	Spicy, cummy, caraway
Cyclohexanone, 5‐methyl‐2‐(1‐methylethylidene)‐, (r)‐	886.2	0.029 ± 0.03	Peppermint, camphor
Umbellulone	902.9	0.001 ± 0.00	Minty, pungent
Cryptone	925.4	0.013 ± 0.01	
2‐acetyl‐3,5‐dimethylpyrazine	933.2	0.080 ± 0.06	Nutty, roasted, hazelnut
Piperitone	976.8	0.075 ± 0.05	Herbal, minty
Ethyl maltol	1,201.5	0.003 ± 0.00	Sweet
5‐methyl‐3,5‐octadien‐2‐one	1,227.5	0.021 ± 0.02	Buttery, woody
7‐Oxabicyclo[4.1.0]heptan‐2‐one, 6‐methyl‐3‐(1‐methylethylidene)‐	1,426.3	0.126 ± 0.18	Herbal, minty
Phenols			
Phenol	1,198.1	0.026 ± 0.01	Sweet, medicinal
Phenol, 5‐methyl‐2‐(1‐methylethyl)‐	1,319.1	0.025 ± 0.07	Aromatic, Sweet, medicinal
Phenol, 2‐methyl‐5‐(1‐methylethyl)‐	1,349.2	0.078 ± 0.08	Pungent
Phenol, 3‐(1‐methylethyl)‐	1,352.3	0.029 ± 0.00	Sweet, medicinal
Phenol, 5‐methyl‐2‐(1‐methylethyl)‐	1,319.1	0.025 ± 0.07	Aromatic, Sweet, medicinal
Sulphur compounds			
Trisulfide, dimethyl	639.9	0.351 ± 0.16	Sulphurous, alliaceous, eggy
Disulfide, methyl (methylthio)methyl	1,085.7	3.578 ± 6.59	Sulphurous, onion
Terpenes/Terpenoids			
Eucalyptol (1,8‐cineole)	423.3	0.895 ± 0.05	Minty, eucalyptoid
Cyclohexene, 1‐methyl‐4‐(1‐methylethylidene)‐	530.4	0.261 ± 0.12	Fresh, sweet, woody, citrus, pine
3,7‐dimethyl‐1,3,6‐octatriene	739.8	0.453 ± 0.08	Woody, tropical, floral
δ‐Elemene	749.3	0.013 ± 0.04	Sweet herbal, woody
Tricyclo[4.4.0.0(2,7)]dec‐3‐ene, 1,3‐dimethyl‐8‐(1‐methylethyl)‐	768.8	1.013 ± 0.94	Woody, spicy, honey
Methacrolein	782.4	0.010 ± 0.01	Floral
Naphthalene, 1,2,3,4,4a,5,6,8a‐octahydro‐7‐methyl‐4‐methylene‐1‐(1‐methylethyl)‐, (1à,4aà,8aà)‐	815.9	0.047 ± 0.02	Fresh, woody
α‐zingiberene	817.6	0.914 ± 0.01	Spice, fresh
*trans*‐à‐Bergamotene	850.1	0.224 ± 0.09	Woody, warm, tea
α‐Santalene	852.8	1.144 ± 0.24	Woody
β‐Caryophyllene	866.2	1.075 ± 1.04	Spicy, peppery, woody
α‐Cubebene	901.7	0.010 ± 0.00	Herbal, earthy
Epi‐β‐santalene	906.3	0.008 ± 0.00	Woody
Terpinyl acetate<delta‐>	918.4	0.014 ± 0.01	Sweet, herbaceous
α‐Caryophyllene	933.7	0.389 ± 0.07	Musty, green
Farnesene<(E)‐β‐	950.1	0.002 ± 0.01	Fruity, woody, citrus, sweet
Bisabolene<(Z)‐α‐	1,028.3	0.089 ± 0.10	Sweet, spicy, balsamic
*cis*‐Carveol	1,095.4	0.070 ± 0.07	Spicy, caraway
α‐Phellandrene	1,099.0	1.993 ± 2.96	Peppery
2,3‐dioxabicyclo[2.2.2]oct‐5‐ene, 1‐methyl‐4‐(1‐methylethyl)‐	1,146.5	0.264 ± 0.18	Fatty, herbaceous
Limonene dioxide	1,152.2	0.065 ± 0.02	Citrus‐like, green
Carvacrol	1,341.5	0.004 ± 0.02	Pungent
Miscellaneous compounds			
Butylhydroxytoluen (BHT)	1,136.8	0.016 ± 0.02	Mild, phenolic, camphor
Tris(methyl thio) methane	1,194.3	0.623 ± 0.87	Earthy, mushroom, musty

Notes. RT: retention time (in s).

a Flavor compounds were identified using the following references (Adams, R. P. 1995. Identification of essential oil components by gas chromatography/mass spectrometry. Allured Publishing Corporation, Carol Stream, IL; Weyerstahl, P., Marschall, H., Thefeld, K., & Subba, G. C. 1998. Constituents of the essential oil from the rhizomes of *Hedychium gardnerianum* Roscoe. Flavour and Fragrance Journal, 13, 377–388; Jirovetz, L., Buchbauer, G., Stoyanova, A. S., Georgiev, E. V., & Damianova, S. T. 2003. Composition, quality control, and antimicrobial activity of the essential oil of long‐time stored dill (*Anethum graveolens* L.) seeds from Bulgaria. Journal of Agricultural and Food Chemistry, 51, 3854–3857; Behera, S., Nagarajan, S., & Rao, L. J. M. 2004. Microwave heating and conventional roasting of cumin seeds (*Cuminum cyminum* L.) and effect on chemical composition of volatiles. Food Chemistry, 87, 25–29; Burdock, G. A. (2010). Fenaroli's handbook of flavor ingredients 6th ed. CRC Press, NW, Boca Raton. http://www.google.com/patents/US4301184; http://www.thegoodscentscompany.com; http://www.perflavory.com).

**Figure 2:**
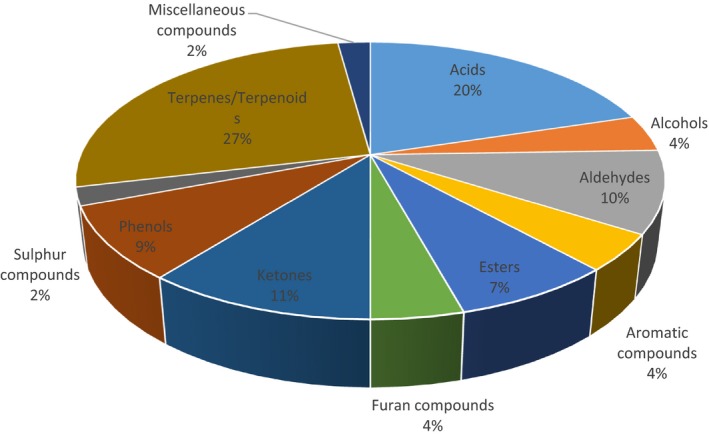
Percentage representation of the flavor compounds in *nkui*

Alcohols are present in plants likely formed through physical damage, during storage, and processing (Eriksson, 1967; Goncalves et al., 2016). The alcohols identified in *nkui* constituted a total of 4% (Figure 2). Of the 14 alcohols recovered, the most abundant one was linalool exhibiting a floral, citrus like aroma. Other significant alcohol compounds found were 1,6‐octadien‐3‐ol, 3,7‐dimethyl‐, acetate, 2,6‐octadien‐1‐ol, 3,7‐dimethyl‐(E)‐ and its ester 2,6‐octadien‐1‐ol, 3,7‐dimethyl‐, acetate contributing a bergamot, floral, and sweet flavor to *nkui* (Table 2). Linalool (Figure 1) is a terpene alcohol which occurs in plants, spices, tree barks and possess anti‐inflammatory and chemoprotective activity (Peana et al., 2002; Ravizza, Gariboldi, Molteni, & Monti, 2008). Its presence in *nkui* could possibly be linked to the therapeutic and estrogenic use of the soup for lactating mothers in Cameroon, as demonstrated in an in vivo study by Tchoupang et al. (2016).

The largest group of compounds found in the *nkui* were terpenes and terpenoids (Figure 2). Terpenes constitute the largest class of natural products and are major sources of natural flavor additives in foods, fragrances, cosmetics, and in alternative medicines (Singh & Sharma, 2015). They are usually endogenous in plants as secondary metabolites and could also be formed through biosynthesis by certain microorganisms. The major compounds in this category were à‐phellandrene (2%) (Figure 1), α‐santalene (1.14%), and β‐caryophyllene (1.07%) (Table 2), all causing a peppery, woody, and spicy flavor to *nkui*. Other terpenes found in *nkui* were α‐zingiberene, *trans*‐à‐bergamotene, α‐caryophyllene, and eucalyptol (Table 2).

According to Takahashi, Sumitani, Inada, and Mori (2002), aldehydes could be formed through the degradation of polyunsaturated fatty acids (PUFA), either by enzymatic action or autoxidation and lipid peroxidation. Few aldehyde compounds were observed in this study (Table 2), with the most significant in terms of % occurrence being 3,7‐dimethyl‐2,6‐octadienal, with a lemon odor. The presence of 3,7‐dimethyl‐2,6‐octadienal has also been reported in several plants and fruits (Burdock, 2010). Similarly, ketone compounds with different chain lengths are abundant in nature and largely contribute to the flavors in plants and spices. Terpenes and their epoxides could possibly react to form ketones and alcohols (Preedy, 2009). Ketones generally occurred in low amounts with relative quantities of 0.63% and 0.46% for 6‐methyl‐5‐hepten‐2‐one and 2‐nonanone, respectively (Table 2). 6‐methyl‐5‐hepten‐2‐one (Figure 1) is an important flavor compound previously reportedly found in plants (Duke, 2000). Other ketone compounds contribution to the overall flavor profile of *nkui* are piperitone, 5‐methyl‐3,5‐octadien‐2‐one and cryptone (Table 2).

Seven esters were identified in this study with linalyl acetate and terpinyl propionate being the predominant ones, with 19% and 1.5%, respectively, found (Table 2). Esters are characterized by pleasant fruit odors that contribute to the aromatic, sweet, and honey notes in foods (Burdock, 2010). Linalyl acetate (Figure 1) is a naturally occurring phytochemical commonly found in spices and plants. It is an ester of linalool having a sweet, green, and citrus smell (Table 2). Its production from linalool has been hypothesized as being catalyzed by an alcohol acetyl transferase and biosynthesis via the nonmevalonate (1‐deoxyxylulose phosphate) terpene pathway (Harada, Ueda, & Iwata, 1985; Zaks, Davidovich‐Rakanati, Bar, Inbar, & Lewinsohn, 2008). Similar to linalool, linalyl acetate have also reported to have anti‐inflammatory, anti‐hypertensive, and analgesic effects (Peana et al., 2002).

Qualitatively, the acids were the sixth most important group of compounds identified in the SPME‐GC×GC‐TOF‐MS analysis of *nkui* (Figure 2). In terms of percentage abundance, the major acid observed was acetic acid (1.7%) known to induce a vinegar, sour and pungent odor. Two sulfur compounds identified in this study were disulfide, methyl (methylthio)methyl (3.6%) and trisulfide, dimethyl (0.35%) conferring a sulphurous and alliaceous smell. Also referred to as 2,3,5‐trithiahexane (TTH), disulfide, methyl (methylthio)methyl has been reported as volatile components in *Brassica* species conferring a sulfury, allecoius odor (Spadone, Matthey‐Doret, & Blank, 2006). Other significant aromatic, bicyclic and furan compounds found were benzene, 1‐methyl‐4‐(1‐methylethyl)‐ (8.2%), 7‐oxabicyclo[4.1.0]heptan‐2‐one, 6‐methyl‐3‐(1‐methylethylidene)‐ (0.13%) and 2‐furanmethanol, 5‐ethenyltetrahydro‐à,à,5‐trimethyl‐, *cis*‐ (0.73%), respectively (Table 2). These compounds influenced the earthy, herbal, spicy, and minty flavor in *nkui*.

## 4 CONCLUSION

The present work represents a comprehensive study on the volatile flavor profile of *nkui*. Using SPME‐GC×GC‐TOF‐MS, this study was able to establish the volatile profile of *nkui* allowing complementary qualitative information. Findings from this study showed the presence of major flavor‐related volatile compounds all contributing to the flavor and aroma of *nkui*. These major compounds were linalyl acetate, linalool, linalyl anthranilate, and 1,6‐octadien‐3‐ol,3,7‐dimethyl, acetate. Such data demonstrate the diverse range of flavors present in *nkui* and could possibly be potential sources of natural flavors for their use in the food and fragrance industries. Nonetheless, it is recommended to quantitatively analyze these volatile compounds, especially the major ones using reference standards and should be explored in future studies.

## ETHICAL REVIEW

This study does not involve any human or animal testing.

## CONFLICT OF INTEREST

The authors declare that they do not have any conflict of interest.
